# Limited stability of Hepatitis B virus RNA in plasma and serum

**DOI:** 10.1038/s41598-024-77329-2

**Published:** 2024-11-07

**Authors:** Valerie Ohlendorf, Birgit Bremer, Lisa Sandmann, Carola Mix, Tammo Tergast, Markus Cornberg, Heiner Wedemeyer, Katja Deterding, Benjamin Maasoumy

**Affiliations:** 1 Department of Gastroenterology, Hepatology, Infectious Diseases and Endocrinology, Hannover Medical School, Hannover, Germany; 2 German Center for Infection Research (DZIF), Partner Site Hannover-Braunschweig, Braunschweig, Germany; 3 Centre for Individualised Infection Medicine (CiiM), A Joint Venture of Helmholtz Centre for Infection Research and Hannover Medical School, Hannover, Germany; 4 Centre for Experimental and Clinical Infection Research, A Joint Venture between the Hanover Medical School and the Helmholtz Centre for Infection Research, TWINCORE, Braunschweig, Germany; 5 Cluster of Excellence RESIST (EXC 2155), Hannover Medical School, Hannover, Germany

**Keywords:** Hepatitis B virus, Pregenomic HBV RNA, Stability, Treatment cessation, Relapse, Hepatitis B, Hepatitis B virus

## Abstract

Pregenomic hepatitis B virus RNA (HBV pgRNA) is a potential biomarker in the management of HBV infected patients. However, prior to the use in routine clinical practice potential confounders of test results need to be identified. This study investigates the stability of HBV pgRNA under various storage conditions. HBV-RNA level of 26 HBV patients were determined using the Roche cobas® 6800/8800 investigational HBV-RNA assay. Plasma and serum were stored for 6,48,169 h at 4,25 and 42 °C, respectively. Additionally, 10 serum and plasma samples underwent 4 or 11 cycles of freezing (−80 °C) and thawing (25 °C). A significant decline in mean pgRNA concentration compared to baseline was observed after storage for 48 h at 25 °C as well as after 6 h of storage at 42 °C. Accordingly, sub-analyses of predefined pgRNA baseline concentrations (≤ 10 cp/mL, > 10–100 cp/ml, > 100 cp/mL) revealed significant changes in pgRNA level after storage at 25 and 42 °C. No effect of freezing and thawing on pgRNA level was observed. A qualitative detection of HBV pgRNA is feasible in samples with > 100 cp/mL up to 48 h under storage temperatures of 4–42 °C. For most stable quantitative HBV pgRNA values storage at 4 °C should be preferred.

## Introduction

Chronic hepatitis B virus infection (CHB) with its long-term complications like liver cirrhosis and hepatocellular carcinoma (HCC) still represents a serious public health problem worldwide^1^.

The current internationally recommended therapy is the oral intake of the nucleos(t)id analogues (NA) tenofovir (TDF) or entecavir (ETV)^2^. NA inhibit the reverse transcriptase activity of the HBV polymerase and lead to virological and biochemical response in the majority of patients and thereby reduce the risk of liver fibrosis and HCC development^3–5^. Nevertheless, NA do not sufficiently suppress de novo synthesis of the nuclear covalently closed circular DNA (cccDNA), which results in a persisting HBV RNA and HBV protein synthesis^6^.

International guidelines consistently suggest therapy continuation until HBs antigen (HBsAg) loss in HBeAg negative patients, while for HBeAg positive patients, guidelines differ between the recommendations for HBsAg loss or HBeAg seroconversion as endpoints for therapy cessation^2^. The latter probably results from the rare functional cure rates (defined as HBV DNA and HBV surface antigen (HBsAg) seroclearance with or without HBsAg seroconversion^7^) during NA treatment^8^. Therefore, long-term or even life-long NA treatment is necessary in most of HBV infected patients. Considering the significant financial burden and the burden of life-long medication intake, surrogate markers that help to estimate the outcome of NA discontinuation before HBsAg loss were studied intensively in the last decade^5,9^.

The remaining cccDNA is believed to be the main reason for viral persistence during NA treatment as well as for viral relapse after cessation of NA treatment^10^. In consequence to the need of liver biopsies for the quantification of the cccDNA level, its role in the therapy management of hepatitis B patients is limited in clinical practice^5^. HBV pregenomic RNA (pgRNA) is described as a promising new serum marker for the identification of persistent cccDNA replication^11^. Since NA’s do not block the formation of HBV-RNA virion-like particles, this biomarker reflects the activity of cccDNA even under NA treatment^12^. Therefore, hepatitis B pgRNA was evaluated in several contexts of HBV infection, including treatment efficacy of drugs affecting RNA transcription, pgRNA stability and pgRNA packaging, or the prediction of ALT flares after therapy cessation^13–15^.

Prior to the possible utilization of HBV RNA as a biomarker in routine medical practice, with longer itineraries and less controlled transport conditions, and to ensure a valid interpretation of existing and future study data potential confounders of test results need to be identified. This study aimed to investigate the stability of HBV RNA from different sample types under various storage conditions. Further, freezing, and thawing effects on HBV RNA levels were investigated.

## Material and methods

This study was conducted in accordance with both the declarations of Helsinki and Istanbul and was approved by the local ethic committee of Hannover Medical School (9227_BO_K_2022). All study participants gave written informed consent for study participation. Clinical laboratory tests were performed according to the Good Clinical Laboratory Practice standards.

### Study cohort

Patients with HBV infection, who were treated in the outpatient clinic of the Department of Gastroenterology, Hepatology, Infectious diseases and Endocrinology of Hannover Medical School, Germany, were recruited for study participation between 1st of October 2020 to 21st of November 2022. Inclusion criteria were age ≥ 18 years, willingness of study participation and HBV infection (defined by quantifiable HBsAg). Patients, who were coinfected with hepatitis C virus (HCV), human immunodeficiency virus (HIV) or hepatitis D virus (HDV) were excluded from the study.

Standard of care laboratory parameters like ALT, HBV DNA level and HBsAg level were analyzed by the local laboratory of Hannover Medical School. HBV DNA levels were determined using the Aptima HBV Quant Assay running on the fully automated Panther® system, following the manufacturer’s protocol (Hologic, Marlborough, Massachusetts, USA). For the assessment of HBsAg level the ARCHITECT HBsAg assay (Abbott, North Chicago, Illinois, USA) was used.

### Blood collection and sample storage before for the assessment of HBV RNA level

**Fig. 1:**
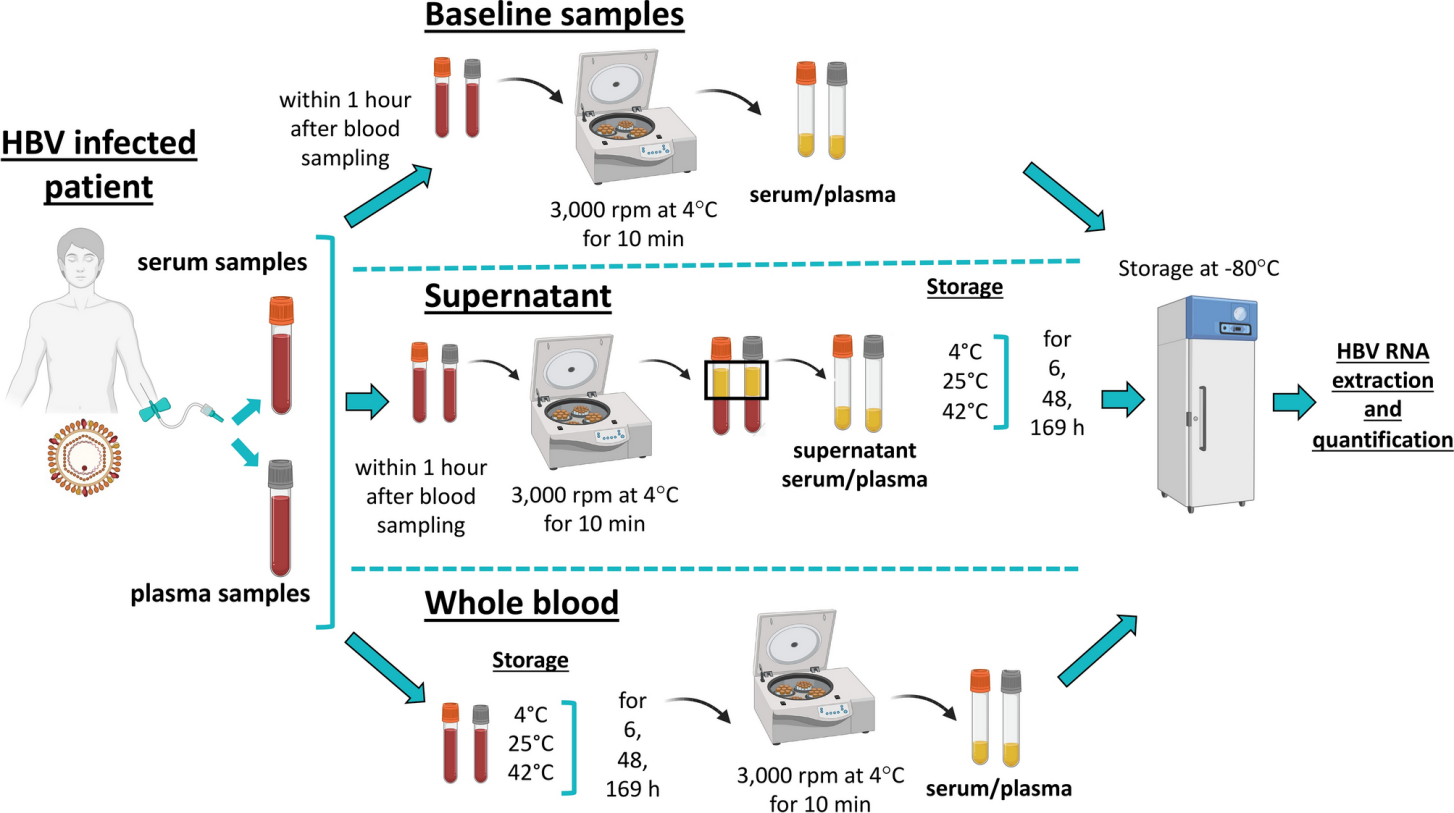
Workflow of blood collection and sample processing of baseline, whole blood and supernatant samples, analyzed in the study.

Blood was collected in plasma (coagulant: ethylenediaminetetraacetate (EDTA)) and serum tubes (clot activator: silicate) (SARSTEDT AG & Co. KG, Nümbrecht, Germany), respectively. Baseline samples (BL) of serum and plasma were centrifuged with 3,000 rpm at 4 °C for 10 min within 1 h (h) after blood collection and afterwards stored at -80 °C until HBV RNA extraction and quantification. To obtain supernatant (S) of serum and plasma, samples were centrifuged (3,000 rpm at 4 °C for 10 min) within 1 h after blood collection. The removed S was stored at 4 °C, room temperature (25 °C) and 42 °C for 6, 48 and 169 h. Whole blood (WB) serum and plasma samples were likewise stored at 4,25 and 42 °C for 6, 48, 169 h. After the respective storage duration S and WB samples were centrifuged in the previous described way and likewise stored at −80 °C until HBV RNA quantification. An illustrated overview of the detailed workflow is given in (Fig. 1).

### Freezing and thawing cycles

Freezing and thawing experiments were performed with samples with quantifiable HBV RNA levels and a sufficient sample volume for further HBV RNA quantification. In total, 10 plasma and 10 serum samples were analyzed. In one freeze-thaw cycle, the sample was stored at -80 °C for at least 16 h, and subsequently thawed for 4 h at room temperature. For each samples set (serum and plasma samples), four or eleven freeze-thaw cycles were performed before quantification of HBV RNA.

### HBV RNA extraction and quantification

Extraction and quantification of serum and plasma HBV RNA was obtained by using the investigational cobas® HBV RNA PCR assay running on the cobas® 6800/8800 system, following the manufacturer’s protocol (Roche Molecular Systems, Pleasanton, California, USA). The lower limit of quantification of HBV-RNA was 10 copies/mL (cp/mL). Detectable but not quantifiable HBV RNA level (≤ 10 cp/mL) were set to 10 cp/mL for statistical analysis.

### Statistical analyses

Statistical analyses were performed using the software GraphPad Prism version 9.1.2 (GraphPad Software, San Diego, California USA). Baseline HBV RNA level, further laboratory parameters as well as clinical parameters were expressed as mean (standard deviation) or median (IQR), respectively. For stability analyses, BL HBV RNA levels were expressed as median (IQR) from BL for each storage duration at each storage temperature, respectively. Samples with undetectable HBV RNA were excluded from the analyses. The Wilcoxon test and fisher’s exact test were performed to determine statistically significant changes in detectable HBV RNA level compared to BL. A p value of < 0.05 was considered to be statistically significant.

Since no defined cut-off value exist that defines stability of HBV RNA, a change of > 0.5 log_10_ cp/mL was assumed to be significant. This cut-off was previously described from Pawlotsky et al.^16^ as a clinically significant change in HCV viremia and was already used by Rattanachaisi et al.^17^ in the context of HBV RNA stability. Therefore, to address the issue of intrinsic assay variability, sub-analyses with cut-off values > 0.5 log_10_ cp/mL change compared to BL were performed. Since the clinically significance of HBV pgRNA changes of > 0.5 log_10_ cp/mL is not clarified for the clinical course of HBV infections, HBV pgRNA changes of > 0.5 log_10_ cp/mL are hereinafter described as “evident stability drop”.

Assuming that storage effects on HBV RNA level as well as detection variabilities are more relevant in samples with low BL HBV RNA level, further subanalyses were performed for 1) not quantifiable HBV RNA level (≤ 10 cp/mL) 2) HBV RNA concentrations of > 10–100 cp/mL and 3) HBV RNA level ≥ 100 cp/mL, respectively. Furthermore, for samples that underwent freezing and thawing experiments, subanalyses were performed for samples with HBV RNA values < 100 cp/mL, 500–5000 cp/mL and > 5000 cp/mL.

## Results

### Characteristics of the study population

From January 2021 to December 2022, 26 patients with HBV infection were included in the study. The median age of the study population was 42 years (34–48.2) and 69% of the subjects (N = 18) were male. The median HBV DNA level at BL was 1 × 10^3^ IU/mL. Further baseline characteristics of the study population are presented in (Table 1).

**Table 1:** Baseline parameters of the study cohort.

		HBV RNA level		
	Total (N = 26)	Not quantifiable (≤ 10 copies/mL) (N = 4/6)^#^	> 10–100 copies/mL (N = 4)	> 100 copies/mL (N = 8)
Male sex	18 (69%)	1/2 (25/33.33%)	3 (75%)	6 (75%)
Age (years)	42 (34–48.25)	39/38 (32–47)	38.5 (29.75–48)	43 (34–44.74)
Fibroscan (kPa)	5.4 (4.4–8.5)	4.6/5.1 (3.8–5.9)	4.65 (4.25–6.4)	9 (6.98–9.6)
NA treatment	15 (57.69%)	2/3 (50%)	1 (25%)	5 (62.50%)
Tenofovir (TDF)	8 (53.33%)	1/2 (25/66.67%)	1 (100%)	2 (40%)
Entecavir (ETV)	7 (46.67%)	1/1 (25/33.33%)	0 (0%)	3 (60%)
Therapy duration (years)	6.2 (0.55–9.73)	3.82/6.02 (2.61–14.57)	2.61	0.56 (0.29–11.19)
ALT U/L	28 (22–43.5)	24.5/25 (20–30.75)	28.5 (16.25–33.25)	58.5 (42–67)
HBsAg IU/mL	7.42 × 10^3^ (1.97 × 10^3^–2.02 × 10^4^)	1.49 × 10^4^/9.10 × 10^3^ (5.37 × 10^3^–2.50 × 10^4^)	3 × 10^3^ (2.10 × 10^3^–6.70 × 10^3^)	2.02 × 10^4^ (4.41 × 10^3^–1.09 × 10^5^)
HBeAg negative	22 (84.62%)	4/6 (100 /100%)	4 (100%)	3 (37.50%)
HBV DNA IU/mL	1 × 10^3^ (0–3 × 10^4^)	5.10 × 10^2^/5 × 10^2^ (0–3.50 × 10^3^)	9 × 10^2^ (2.15 × 10^2^–7.75 × 10^3^)	2.05 × 10^6^ (2.48 × 10^41^–1.60 × 10^8^)

Continuous values are expressed in median (IQR). Categorical values are depicted as number and frequency.

^#^Numbers of patients with not quantifiable serum/ plasma samples.

### HBV RNA level in baseline samples

HBV RNA was detected in 61.5% (N = 16) of serum and 69.2% (N = 18) of plasma samples (p = 0.77), respectively. Detectable baseline HBV RNA levels did not differ significantly between serum (median = 1.23 × 10^2^ cp/mL, 1.04 × 10^1^–2.46 × 10^5^cp/mL) and plasma samples (median = 2.19 × 10^1^ cp/mL, 1.00 × 10^1^–9.62 × 10^4^cp/mL) (p = 0.21).

The distribution of samples with detectable HBV RNA and distribution of samples with 1) not quantifiable HBV RNA levels, 2) levels between > 10–100 cp/mL, and 3) HBV RNA level > 100 cp/mL as well as median and mean HBV RNA level in serum and plasma samples are presented in (Table 2).

**Table 2:** HBV RNA concentrations in serum and plasma samples at baseline.

	Plasma samples	Serum samples
	(N = 26)	(N = 26)
Tnd*	8 (30.77)	10 (38.46)
not quantifiable HBV RNA level (≤ titer min)	6 (23.08)	4 (15.39)
Quantifiable HBV RNA level	12 (46.15)	12 (46.15)
> 10 -100 copies/mL	4 (33.33.39)	4 (33.33)
> 100 copies/mL	8 (66.67)	8 (66.67)
HBV RNA copies/mL (median)	21.90 (10–1.75 × 10^3^)	122.70 (10–2.01 × 10^3^)
HBV RNA copies/mL (mean)	4.63 × 10^6^ (1.75 × 10^7^)	5.35 × 10^6^ (1.90 × 10^7^)

Continuous values are expressed as median with (IQR) or mean with (standard deviation), respectively. Categorical values are depicted as number and frequency.

*tnd = target not detected; < titer min = titer below the LLoQ.

### Analyses of mean HBV RNA level after various storage conditions

#### Storage at 4 °C

Mean HBV RNA level in whole blood plasma and serum samples remained stable until 48 h of storage at 4 °C (plasma: BL = 4.63 × 10^6^ cp/mL, 48 h = 5.04 × 10^6^ cp/mL [p = 0.77]; serum: BL = 5.35 × 10^6^ cp/mL, 48 h = 5.77 × 10^6^ cp/mL [p = 0.17]). Longer storage up to 169 h at 4 °C resulted in a slight but not statistically significant decrease in mean HBV RNA levels in these sample types (plasma: BL = 4.63 × 10^6^ cp/mL, 169 h = 4.55 × 10^6^ cp/mL [p = 0.46]; serum: BL = 5.35 × 10^6^ cp/mL, 169 h = 4.91 × 10^6^ cp/mL [p = 0.12]).

#### Storage at 25 °C

Under storage at 25 °C HBV RNA levels significantly decreased after 48 h of storage in WB plasma samples (BL = 4.63 × 10^6^ cp/mL, 48 h = 3.74 × 10^6^ cp/mL, p = 0.005). After 169 h a significant decline in HBV RNA level compared to BL was detected in all sample types (WB plasma: 169 h = 4.35 × 10^6^ cp/mL [p = 0.038], WB serum: 169 h = 4.14 × 10^6^ cp/mL [p < 0.0001]; supernatant plasma: 169 h = 3.44 × 10^6^ cp/mL [p < 0.0001]; supernatant serum: 169 h = 2.73 × 10^6^ cp/mL [p = 0.0002]).

#### Storage at 42 °C

**Fig. 2:**
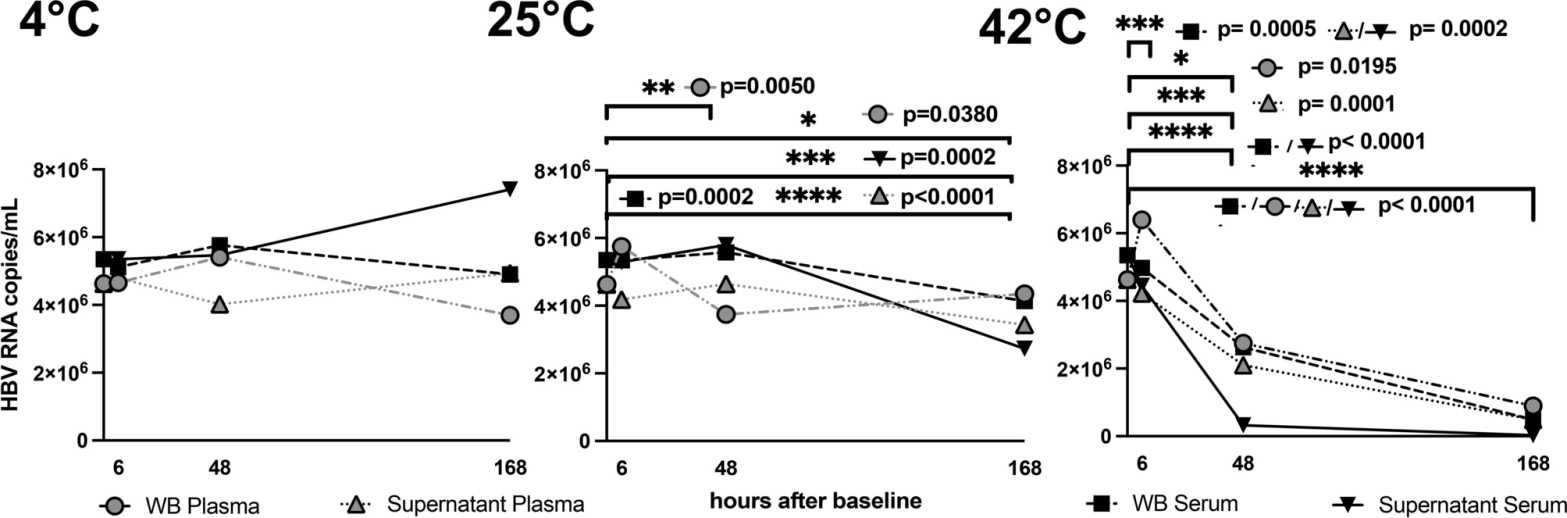
Mean HBV RNA (copies/mL) levels in supernatant and whole blood of serum and plasma samples after storage for 6, 48 and 169 h at 4, 25 and 42 °C.

In contrast, under a storage temperature of 42 °C, a statistically significant decline in mean HBV RNA occurred already after 6 h of storage (WB serum: BL = 5.35 × 10^6^ cp/mL, 6 h = 4.97 × 10^6^ cp/mL [p = 0.0005]; supernatant plasma: BL = 4.63 × 10^6^ cp/mL, 6 h = 4.23 × 10^6^ cp/mL [p = 0.0002]; supernatant serum: BL = 5.35 × 10^6^ cp/mL, 6 h = 4.45 × 10^6^ cp/mL [p = 0.0002]). (Fig. 2).

#### Evident stability drops in mean HBV RNA

To address the aspect of intrinsic assay variability, subanalyses with cut-off values > 0.5 log_10_ cp/mL change compared to BL were performed. These sub-analyses yielded a clinically and statistically significant decline compared to baseline exclusively in samples stored at 42 °C for 48–169 h (WB plasma: BL = 2.83 log_10_ cp/mL, 48 h = −0.58 log_10_ cp/mL [p = 0.0195], 169 h = −0.86 log_10_ cp/mL [p < 0.0001]; WB serum: BL = 3.02 log_10_ cp/mL, 48 h = −0.63 cp/mL [p < 0.0001], 169 h = −1.06 log_10_ cp/mL [p < 0.0001]; supernatant plasma: BL = 2.83 log_10_ cp/mL, 169 h = −0.82 log_10_ cp/mL [p < 0.0001]; supernatant serum: BL = 3.02 log_10_ cp/mL, 48 h = −0.80 log_10_ cp/mL [p < 0.0001], 169 h = −1.36 log_10_ cp/mL [p < 0.0001]).

### Stability of various HBV RNA concentrations under various storage conditions

To investigate storage effects on qualitative HBV RNA results, subanalysis were performed in samples with different HBV RNA levels, 1) not quantifiable HBV RNA level (≤ 10 cp/mL) 2) HBV RNA concentrations of > 10–100 cp/mL and 3) HBV RNA level > 100 cp/mL, respectively.

#### Stability of not quantifiable HBV RNA (≤ 10 cp/mL)

Not quantifiable HBV RNA level occurred in 33.33% (N = 6/18,) of plasma and 25% (N = 4/16,) of serum samples (p = 0.71). Statistically significant changes in the number of samples with detectable HBV RNA were present after 169 h of storage at 25 °C in whole blood samples (BL N = 6, 169 h N = 1, 16.67%, p = 0.0156) as well as at 42 °C in whole blood samples and in supernatant of both plasma and serum samples after 48 h of storage (WB plasma: BL N = 4, 48 h N = 0, 169 h N = 0, 0%, p = 0.0022, WB serum: BL N = 4, 48 h N = 0, 169 h N = 0, 0%, p = 0.0286; supernatant plasma: BL N = 6, 169 h N = 1, 16.67%, p = 0.0152; supernatant serum: BL N = 4, 169 h N = 0, 0%, p = 0.0286).

#### Stability of HBV RNA concentrations of > 10–100 cp/mL

HBV RNA levels of > 10–100 cp/mL were detected in 22.22% (N = 4/18, median = 1.55 × 10^1^ cp/mL) of plasma and 25% (N = 4/16, median = 2.58 × 10^1^ cp/mL) of serum samples (p > 0.99). A significant decrease in HBV RNA to undetectable HBV RNA was obtained in whole blood serum (BL median = 1.95 × 10^1^ cp/mL) and plasma samples (BL median = 1.55 × 10^1^ cp/mL) stored at 42 °C after 48–169 h of storage (p = 0.0286).

#### Stability of HBV RNA concentrations of > 100 cp/mL

In samples with HBV RNA level > 100 cp/mL (plasma: 44.44%, N = 8/18, median = 1.55 × 10^5^ cp/mL; serum: 50%, N = 8/16, median = 1.78 × 10^5^ cp/mL, p > 0.99) a significant decline in HBV RNA level was detected 48–169 h after BL under storage at 25 °C (48 h: WB plasma samples −3.34 × 10^4^ cp/mL, p = 0.0391; 169 h: WB serum samples −4.04 × 10^4^ cp/mL, p = 0.0078, supernatant plasma samples −5.40 × 10^4^ cp/mL, p = 0.0078, and supernatant serum samples −1.04 × 10^5^ cp/mL, p = 0.0078), respectively. At a storage temperature of 42 °C, a statistically significant decline in HBV RNA was already obtained after 6 h in WB serum samples (−1.82 × 10^4^ cp/mL, p = 0.0078), supernatant plasma samples (−3.35 × 10^3^ cp/mL, p = 0.0078), and supernatant serum samples (−4.62 × 10^4^ cp/mL, p = 0.0078), respectively.

#### Evident stability drop in various HBV RNA concentrations under various conditions

An evident stability drop (> 0.5 log_10_) in median HBV RNA compared to BL within the subgroups of the investigated HBV RNA cut-offs was detected in whole blood serum samples with concentrations of > 10–100 cp/mL stored at 4 °C for 169 h (BL = 1.27 log_10_ cp/mL, 169 h = −0.54 log_10_ cp/mL, p = 0.88) as well as supernatant serum samples stored for 6 h (BL = 1.27 log_10_ cp/mL, 6 h = −0.74 log_10_ cp/mL, p = 0.25) or 48 h (BL = 1.27 log_10_ cp/mL, 48 h = -0.60 log_10_ cp/mL p = 0.13), respectively. In detail, in 1/4 (25%) plasma WB as well as in 1/4 (25%) S sample with concentrations of > 10–100 cp/mL an evident stability drop was observed, while this was the case in 2/4 (50%) serum WB and S samples within the respective concentration range.

At 25 °C, an evident stability drop in median HBV RNA level occurred exclusively in the subgroup of samples with HBV RNA values of > 10–100 cp/mL after 169 h of storage (WB plasma samples −0.57 log10_10_ cp/mL, p = 0.38; WB serum samples −1.09 log10_10_ cp/mL, p = 0.25).

Under this storage temperature in 2/4 (50%) of WB plasma and 1/4 (25%) S plasma samples with an HBV RNA concentration of > 10–100 cp/mL an evident stability drop was observed. In serum samples with comparable HBV RNA concentrations 3/4 (75%) WB and 2/4 (50%) S samples showed an evident stability drop.

In contrast, under storage at 42 °C, an evident stability drop of median HBV RNA levels occurred in all sample types independent of the HBV RNA value range. Notably, in whole blood serum samples with HBV RNA levels between > 10–100 cp/mL an evident stability drop in HBV RNA level already occurred after 6 h of storage (BL = 1.27 log_10_ cp/mL, 6 h = −0.60 log_10_ cp/mL, p = 0.25). Within samples with > 10–100 cp of HBV RNA/mL 100% (4/4) of WB plasma and serum samples showed an evident stability drop, while the frequency was lower in the respective S samples (plasma: 2/4, 50%; serum: 3/4, 75%). Under this storage temperature also in samples with HBV RNA concentrations > 100 cp/mL a high rate of significant stability drops was observed (plasma: WB N = 6/8, 75%, S N = 8/8, 100%; serum: WB N = 7/8, 87.50%, S N = 8/8, 100%).

An overview of the log_10_ change of median HBV RNA values of the respective subgroups as well as of individual HBV RNA values according to the respective storage temperatures is given in suppl. table 1–3, respectively. Additionally, suppl. table 4 shows the percentage changes in median HBV RNA level under the respective storage conditions.

### HBV RNA levels after multiple freezing and thawing cycles

Median HBV RNA level in plasma samples (BL = 2.90 × 10^4^ cp/mL) remained stable even after eleven cycles of freezing and thawing (median HBV RNA level = 2.43 × 10^4^ cp/mL, p = 0.49). The same trend was observed for serum samples: median BL = 5.17 × 10^3^ cp/mL and median = 5.57 × 10^3^ cp/mL after eleven cycles of freezing and thawing (p = 0.07), respectively.

As the investigated samples showed high differences in the HBV RNA concentration, subanalyses were performed of samples with HBV RNA values < 100 cp/mL, 500 – 5000 cp/mL and > 5000 cp/mL.

#### Stability of predefined HBV RNA levels after multiple freezing and thawing cycles

**Fig. 3:**
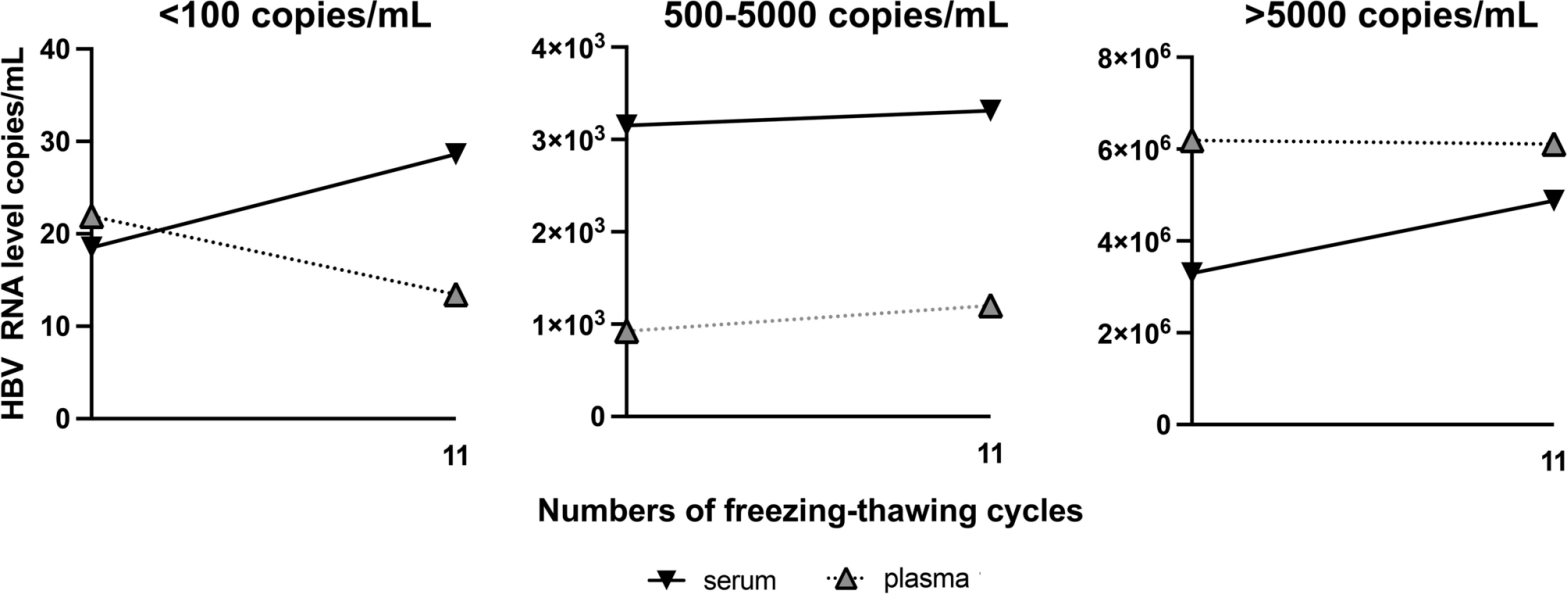
Median HBV RNA (copies/mL) in serum and plasma samples after 11 cycles of freezing and thawing.

A slight decrease of HBV RNA levels was detected in samples with HBV RNA levels < 100 cp/mL (range: 0.56 × 10^1^ to 1.28 × 10^1^ cp/mL) after 11 cycles of freezing and thawing, except of one serum sample where a slight increase (1.16 × 10^1^ cp/mL) in the HBV RNA level after 11 cycles of freezing and thawing was observed. In the majority of samples with BL HBV RNA concentrations between 500 and 5000 cp/mL as well as in samples with HBV RNA concentrations > 5000 cp/mL, HBV RNA levels remained stable even after eleven cycles of freezing and thawing over all sample types (Fig. 3).

Of note, an evident stability drop of HBV RNA occurred exclusively in one serum sample with HBV RNA level < 100 cp/mL, that became undetectable after 4 cycles of freezing and thawing (suppl. Figure 1).

## Discussion

Pregenomic HBV RNA is intensively analyzed as a biomarker in the management of HBV infected patients. Over the last years, several studies investigated the value of HBV RNA in clinical contexts, including therapy monitoring (NA as well as interferon), therapy cessation and the risk of ALT flares after treatment cessation^15,18–20^.

HBV RNA is transcribed from cccDNA that is converted out of relaxed circular HBV DNA after viral entry in the hepatocyte^21^. Five kinds of HBV RNAs are known, including the 3.5-kb precore mRNA (pcRNA), pgRNA, 2.4/2.1-kb surface mRNA and 0.7 kb X mRNA^22,23^. In blood, pgRNA is considered to be the predominant HBV RNA species, but it remains arguable in which proportion the known forms (3.5-kb pgRNA, 3’truncated pgRNA or spliced pgRNA) circulate^24,25^.

So far, no standardized assay for HBV pgRNA with optimized specificity and sensitivity exists. Assays vary about different types of quantitative PCR methods, but no standardized amplification target is defined. As different types of HBV pgRNA may be detected with the respective assays, a comparison of study results remains challenging. Therefore, a standardized HBV RNA assay to achieve generalized HBV RNA quantification is highly needed^25^. However, next to the need of a standardization of assays, for a comparability of study results knowledge about the stability of circulating HBV RNA is necessary.

In this study we investigated the stability of different HBV RNA concentrations in supernatant and whole blood collected in plasma and serum tubes that were stored for 6, 48 or 169 h at 4°, 25 and 42 °C, respectively. Our results revealed stable HBV pgRNA concentrations > 100 cp/mL in serum and plasma samples stored at 4 °C. Under these conditions, no statistically significant impact of storage on HBV RNA levels and no evident stability drop (> 0.5 log_10_ cp/mL) was observed. Under storage of 25 °C, within the subgroup of samples with HBV RNA concentrations > 100 cp/mL, a statistically significant decline in all sample types occurred after 48–169 h of storage. At storage temperature of 42 °C, a statistically significant decline of HBV RNA levels was additionally observed in samples with initial HBV RNA concentrations of ≤ 10 cp/mL as well as > 10–100 cp/mL. Of note, also differences in the stability of HBV pgRNA between whole blood and supernatant samples were observed. Under storage at 25 °C a statistically significant decline in number of samples with HBV RNA levels < 10cp/mL occurred exclusively in whole blood plasma samples. In contrast, under storage at 42 °C no statistically significant decline in HBV RNA levels was observed in supernatant plasma and serum samples with baseline concentrations between > 10–100 cp/mL, while HBV RNA was undetectable after 48–169 h after storage in the respective whole blood samples. However, a study including higher numbers of samples is necessary to draw firm conclusions about stability differences between the different sample types. In freezing and thawing experiments no significant effect on HBV RNA level was observed.

To our knowledge, only two studies have investigated the stability of HBV RNA, so far. Rattanachaisit et al.^17^ measured HBV pgRNA levels after storage at 4 and 25 °C for 2, 6, 12, 24 and 48 h in forty plasma samples, respectively. The mean concentration at baseline was 5.58 log_10_ cp/mL with a range between 3.08 to 8.04 log_10_ cp/mL. In this study HBV RNA levels decreased over time at both storage temperatures but did not exceed an evident stability drop of > 0.5 log_10_ cp/mL. The same results were obtained when HBV pgRNA underwent freezing and thawing cycles at −80 and −20 °C. The second study conducted by Anderson et al.^26^ investigated the stability of samples with high HBV pgRNA concentrations (5.5 log_10_ U/mL and 3 log_10_ U/mL). Decrease of HBV pgRNA levels remained < 0.25 log U/mL after storage at 4 °C up to 30 days and at 25–37 °C up to 7 days, respectively. Stable concentrations of HBV pgRNA were additionally obtained after one to three freeze-thaw cycles at −80 °C. In contrast to the presented study, in none of the previously performed stability analyses, HBV pgRNA concentrations ≤ 100 cp/mL were investigated. Considering the impact of low HBV pgRNA levels in the context of clinical decisions, our performed subanalyses of the stability of HBV pgRNA of different concentration ranges revealed important findings in the handling of these samples. For example, Seto et al.^15^ as well as Fan et al.^27^ postulated, that undetectable HBV RNA levels at end of treatment, combined with levels of HBsAg or HBcrAg, could be used for a safe NA cessation with low risks of clinical relapse. If this study results should be included in clinical practice guidelines, it remains mandatory that negative HBV RNA levels reflect the individual natural history of HBV infection and are not caused by storage or transport conditions. Furthermore, stability of HBV RNA was previously not investigated at storage temperatures of 42 °C. Assuming that in daily clinical practice blood samples are sometimes stored at places under direct sunlight (for example accidental storage of blood samples on windowsills) or for blood samples drawn from patients living in tropical low-income countries with commonly occurring electrical blackouts, the effect of temperatures > 40 °C on the stability of HBV pgRNA is also of interest. However, since a significant decline in HBV RNA already occurred after 48 h under storage at 42 °C it is of high interest to evaluate a time range in which it is possible to detect and quantify HBV-RNA in samples stored at 42 °C. Importantly, in the presented study, samples were stored at 42 °C in an incubator to exclude additional effects of UV light on HBV RNA concentrations. In general, higher temperatures affect RNA by temperature-induced reduction in base-pairing, resulting in changes of conformation, as well as by hydrolyzing the RNA, resulting in irreversible damage of RNA integrity^28^. However, even if the exact mechanism of degradation of HBV pgRNA has not been investigated so far, our results support the general knowledge of decreasing RNA concentrations under increasing temperatures in non-thermophilic organisms.

In this study, quantification of HBV RNA was performed using the investigational Cobas® 6800/8800 HBV RNA assay. This assay is based on a fully automated sample preparation and therefore is less error-prone to frequent errors like pipetting inaccuracies. The sequence of the primers is not published in the manual of the assay. However, the manufacturer stated that selective amplification of target pregenomic HBV RNA is achieved by the use of organism-specific primers that hybridize to highly conserved regions of HBV pgRNA. Therefore, as one limitation of this study, we cannot specify the form of HBV pgRNA (3’-truncated vs 3.5-kb HBV RNA) that was quantified with the assay. In consequence, the results of our study are not specific for a single form of HBV pgRNA. Additionally, stability of HBV pgRNA is exclusively reliable for the investigated conditions. The applied assay does not run replicates of blood samples, but the results of test samples with defined HBV RNA concentrations and negative controls, yielded an intra-assay coefficient of variation of 1.03%. Therefore, an excellent reproducibility is assumed for the assay. These results are in line with a study conducted by Scholtès et al.^29^, who evaluated the performance of the HBV RNA cobas® 6800/8800 assay and found coefficients of variations below 5%. The linearity of the assay was evaluated in this study in a range between 10 and 10^7^ cp/mL. Therefore, false negative test results in the subgroup of samples with HBV pgRNA concentrations < 10 cp/mL are feasible. However, since the number of samples with detectable HBV RNA decreased with increasing storage temperature in all sample types, a trend of increasing instability in HBV pgRNA concentrations < 10 cp/mL is clearly visible. As a further limitation of our study, it is noteworthy that RNA integrity was not tested. Therefore, we cannot provide information about the degradation grade of the analyzed samples. Furthermore, to our knowledge it is not known if a prolonged storage time at −80 °C can affect HBV RNA quantification. The mean storage time of our samples was 348 days. Since no quantification of HBV RNA in fresh samples was performed, RNA degradation cannot be excluded.

In conclusion, our study results suggest that a qualitative detection of HBV RNA is feasible in samples with > 100 cp/mL up to 48 h under storage temperatures of 4–42 °C. For a valid interpretation of quantitative HBV RNA values storage at 4 °C should be preferred.

## Supplementary Information

Supplementary Information.

## Data Availability

Data is provided within the manuscript or supplementary information files.

## Abbreviations

HBV: Hepatitis B virus
pgRNA: Pregenomic hepatitis B virus RNA
CHB: Chronic hepatitis B virus infection
HCC: Hepatocellular carcinoma
NA: Nucleos(t)id analogue
TDF: Tenofovir
ETV: Entecavir
cccDNA: Covalently closed circular DNA
HBsAg: HBs antigen
HCV: Hepatitis C virus
HIV: Human immunodeficiency virus
HDV: Hepatitis D virus
ALT: Alanine transaminase
EDTA: Ethylenediaminetetraacetate (EDTA)
BL: Baseline
WB: Whole blood
S: Supernatant
