# Supplementary material for: Limited stability of Hepatitis B virus RNA in plasma and serum
Source: Sci Rep. 2024 Nov 7;14:27128. doi: 10.1038/s41598-024-77329-2 (PMC11543676; doi:10.1038/s41598-024-77329-2)
Supplement: Supplementary file 1 — Supplementary Information. [file 41598_2024_77329_MOESM1_ESM.docx]

**Supplement tables**

**Suppl. table 1** Changes in log HBV-RNA level of individual samples after storage for 6, 48 or 169 h at 4°C compared to baseline. Changes in concentration > log 0.5 are highlighted in black.

| **4°C** | |  | | **Whole blood** | | | | | **Supernatant** | | | | | |
| --- | --- | --- | --- | --- | --- | --- | --- | --- | --- | --- | --- | --- | --- | --- |
|  |  | | **BL** | **6 h** | **48 h** | | **169 h** | | | **6 h** | **48 h** | | **169 h** | |
| **Plasma** | **> 10 – 100 copies/mL** | | **1.19** | **-1.19** | -0.19 | | -0.19 | | | -0.19 | -0.19 | | 0.18 | |
|  |  | | **1.09** | -0.09 | -0.09 | | -0.09 | | | -0.09 | -0.09 | | **-1.09** | |
|  |  | | **1.45** | 0.03 | 0.07 | | 0.17 | | | 0.03 | -0.13 | | 0.36 | |
|  |  | | **1.19** | 0.09 | -0.19 | | -0.19 | | | -0.06 | -0.19 | | -0.08 | |
|  | **median** | | **1.19** | -0.03 | -0.14 | | -0.14 | | | -0.08 | -0.16 | | 0.05 | |
|  | **> 100 copies/mL** | | **2.97** | 0.00 | -0.02 | | -0.06 | | | -0.09 | -0.05 | | -0.15 | |
|  |  | | **3.63** | 0.10 | 0.02 | | -0.04 | | | 0.07 | -0.02 | | -0.13 | |
|  |  | | **5.44** | 0.01 | 0.01 | | -0.01 | | | -0.07 | -0.03 | | -0.08 | |
|  |  | | **7.87** | 0.00 | 0.07 | | -0.11 | | | 0.01 | -0.07 | | 0.03 | |
|  |  | | **6.79** | -0.03 | 0.07 | | 0.03 | | | 0.06 | 0.00 | | -0.01 | |
|  |  | | **4.57** | -0.07 | -0.02 | | 0.03 | | | 0.01 | -0.12 | | -0.04 | |
|  |  | | **6.36** | 0.02 | -0.04 | | 0.02 | | | -0.09 | -0.07 | | -0.04 | |
|  |  | | **2.23** | -0.14 | 0.02 | | -0.01 | | | 0.03 | 0.00 | | 0.02 | |
|  | **median** | | **5.01** | 0.00 | 0.02 | | -0.01 | | | 0.01 | -0.04 | | -0.04 | |
| **Serum** | **> 10 – 100 copies/mL** | | **1.66** | 0.18 | -0.01 | | -0.02 | | | 0.03 | -0.13 | | -0.15 | |
|  |  | | **1.41** | -0.11 | -0.18 | | 0.23 | | | -0.41 | 0.05 | | -0.17 | |
|  |  | | **1.12** | -0.12 | -0.12 | | **-1.12** | | | **-1.12** | **-1.12** | | -0.12 | |
|  |  | | **1.06** | **-1.06** | **-1.06** | | **-1.06** | | | **-1.06** | **-1.06** | | -0.06 | |
|  | **median** | | **1.27** | -0.12 | -0.15 | | **-0.54** | | | **-0.74** | **-0.60** | | -0.14 | |
|  | **> 100 copies/mL** | | **3.01** | -0.03 | 0.01 | | -0.09 | | | -0.06 | -0.05 | | 0.03 | |
|  |  | | **3.70** | 0.03 | 0.00 | | -0.01 | | | 0.06 | 0.02 | | -0.10 | |
|  |  | | **5.50** | -0.09 | 0.06 | | -0.08 | | | -0.05 | -0.10 | | -0.09 | |
|  |  | | **7.88** | -0.02 | 0.03 | | -0.04 | | | 0.01 | 0.16 | | 0.00 | |
|  |  | | **6.84** | -0.04 | 0.00 | | -0.02 | | | -0.03 | -0.06 | | 0.00 | |
|  |  | | **4.60** | -0.03 | 0.09 | | 0.01 | | | 0.01 | 0.04 | | -0.05 | |
|  |  | | **6.33** | 0.02 | 0.07 | | 0.00 | | | 0.03 | 0.00 | | -0.04 | |
|  |  | | **2.30** | -0.06 | 0.03 | | 0.05 | | | -0.07 | -0.04 | | -0.11 | |
|  | **median** | | **5.05** | -0.03 | 0.03 | | -0.02 | | | -0.01 | -0.02 | | -0.05 | |
|  |  | |  |  | |  | |  |  | | |  | |  |

**Suppl. table 2** Changes in log HBV-RNA level of individual samples after storage for 6, 48 or 169 h at 25°C compared to baseline. Changes in concentration > log 0.5 are highlighted in black.

| **25°C** |  |  | **Whole blood** | | | **Supernatant** | | | |
| --- | --- | --- | --- | --- | --- | --- | --- | --- | --- |
|  |  | **BL** | **6 h** | **48 h** | **169 h** | | **6 h** | **48 h** | **169 h** |
| **Plasma** | **> 10 - 100 copies/mL** | **1.19** | -0.19 | -0.19 | **-1.19** | | 0.14 | -0.19 | -0.19 |
|  |  | **1.09** | -0.04 | -0.09 | **-1.09** | | **-1.09** | -0.09 | -0.09 |
|  |  | **1.45** | -0.20 | 0.03 | -0.05 | | 0.19 | 0.17 | -0.01 |
|  |  | **1.19** | -0.06 | -0.12 | 0.09 | | 0.08 | -0.18 | -0.19 |
|  | **median** | **1.19** | -0.13 | -0.11 | **-0.57** | | 0.11 | -0.14 | -0.14 |
|  | **> 100 copies/mL** | **2.97** | -0.04 | -0.11 | -0.09 | | -0.09 | -0.16 | -0.15 |
|  |  | **3.63** | -0.03 | 0.05 | 0.14 | | -0.05 | -0.07 | -0.09 |
|  |  | **5.44** | 0.00 | -0.12 | -0.14 | | -0.07 | -0.09 | -0.12 |
|  |  | **7.87** | 0.11 | -0.10 | -0.02 | | -0.05 | 0.01 | -0.13 |
|  |  | **6.79** | 0.00 | -0.01 | -0.07 | | 0.05 | -0.04 | -0.14 |
|  |  | **4.57** | -0.06 | -0.03 | 0.01 | | -0.05 | -0.10 | -0.20 |
|  |  | **6.36** | -0.04 | -0.12 | -0.10 | | -0.08 | -0.06 | -0.14 |
|  |  | **2.23** | 0.01 | -0.11 | -0.03 | | -0.07 | -0.05 | -0.34 |
|  | **median** | **5.01** | -0.02 | -0.11 | -0.05 | | -0.06 | -0.07 | -0.14 |
| **Serum** | **> 10 - 100 copies/mL** | **1.12** | 0.15 | -0.12 | **-1.12** | | **-1.12** | -0.12 | **-1.12** |
|  |  | **1.66** | 0.08 | -0.12 | 0.08 | | -0.07 | -0.06 | -0.10 |
|  |  | **1.41** | -0.19 | -0.41 | **-1.41** | | -0.11 | -0.11 | -0.41 |
|  |  | **1.06** | **-1.06** | **-1.06** | **-1.06** | | 0.28 | **-1.06** | -0.06 |
|  | **median** | **1.27** | -0.06 | -0.27 | **-1.09** | | -0.09 | -0.12 | -0.26 |
|  | **> 100 copies/mL** | **3.01** | 0.00 | -0.03 | -0.05 | | 0.06 | -0.10 | -0.17 |
|  |  | **3.70** | 0.00 | -0.08 | -0.19 | | -0.01 | -0.21 | -0.46 |
|  |  | **5.50** | 0.00 | -0.07 | -0.15 | | -0.02 | -0.14 | -0.37 |
|  |  | **7.88** | 0.00 | 0.03 | -0.10 | | 0.00 | 0.05 | -0.29 |
|  |  | **6.84** | -0.03 | -0.08 | -0.22 | | -0.09 | -0.15 | -0.32 |
|  |  | **4.60** | -0.04 | -0.06 | -0.19 | | -0.03 | -0.17 | -0.47 |
|  |  | **6.33** | 0.03 | -0.11 | -0.05 | | -0.02 | -0.04 | -0.26 |
|  |  | **2.30** | -0.03 | -0.12 | -0.04 | | -0.02 | -0.03 | -0.27 |
|  | **median** | **5.05** | 0.00 | -0.08 | -0.13 | | -0.02 | -0.12 | -0.31 |

**Suppl. table 3** Changes in log HBV-RNA level of individual samples after storage for 6, 48 or 169 h at 42°C compared to baseline. Changes in concentration > log 0.5 are highlighted in black.

| **42°C** |  |  | | **Whole blood** | | | | **Supernatant** | | |
| --- | --- | --- | --- | --- | --- | --- | --- | --- | --- | --- |
|  |  | **BL** | **6 h** | | **48 h** | **169 h** | **6 h** | | **48 h** | **169 h** |
| **Plasma** | **> 10 - 100 copies/mL** | **1.19** | -0.19 | | **-1.19** | **-1.19** | -0.19 | | **-0.19** | **-0.19** |
|  |  | **1.09** | **-1.09** | | **-1.09** | **-1.09** | -0.09 | | -0.09 | **-1.09** |
|  |  | **1.45** | -0.10 | | 0.39 | **-1.45** | -0.12 | | -0.03 | -0.45 |
|  |  | **1.19** | -0.08 | | **-1.19** | **-1.19** | -0.02 | | -0.16 | -0.19 |
|  | **median** | **1.19** | -0.15 | | **-1.14** | **-1.19** | -0.11 | | -0.13 | -0.32 |
|  | **> 100 copies/mL** | **2.97** | -0.14 | | -0.16 | -0.33 | -0.16 | | -0.23 | **-0.51** |
|  |  | **3.63** | 0.01 | | 0.01 | **-0.57** | -0.08 | | -0.06 | **-1.29** |
|  |  | **5.44** | 0.00 | | -0.24 | -0.44 | -0.05 | | -0.35 | **-0.59** |
|  |  | **7.87** | 0.15 | | -0.23 | **-0.73** | -0.04 | | -0.35 | **-1.00** |
|  |  | **6.79** | 0.03 | | -0.20 | **-0.61** | -0.08 | | -0.31 | **-0.82** |
|  |  | **4.57** | -0.10 | | -0.16 | **-0.53** | -0.08 | | -0.36 | **-1.43** |
|  |  | **6.36** | -0.02 | | -0.25 | **-0.55** | -0.04 | | -0.26 | **-0.73** |
|  |  | **2.23** | -0.05 | | 0.10 | **-0.54** | -0.05 | | -0.18 | **-1.23** |
|  | **median** | **5.01** | -0.01 | | -0.18 | **-0.55** | -0.07 | | -0.29 | **-0.91** |
| **Serum** | **> 10 - 100 copies/mL** | **1.66** | -0.13 | | **-1.66** | **-1.66** | -0.43 | | **-0.61** | **-0.57** |
|  |  | **1.41** | 0.06 | | **-1.41** | **-1.41** | -0.41 | | -0.41 | -0.41 |
|  |  | **1.12** | **-1.12** | | **-1.12** | **-1.12** | **-0.12** | | **-0.12** | **-1.12** |
|  |  | **1.06** | **-1.06** | | **-1.06** | **-1.06** | **-1.06** | | **-1.06** | **-1.06** |
|  | **median** | **1.27** | **-0.60** | | **-1.27** | **-1.27** | -0.42 | | -0.51 | **-0.82** |
|  | **> 100 copies/mL** | **3.01** | -0.01 | | -0.13 | -0.48 | -0.15 | | **-0.50** | **-1.08** |
|  |  | **3.70** | -0.11 | | -0.17 | **-1.69** | -0.10 | | **-1.20** | **-2.50** |
|  |  | **5.50** | -0.09 | | -0.23 | **-0.77** | -0.13 | | **-0.63** | **-1.12** |
|  |  | **7.88** | -0.03 | | -0.32 | **-1.07** | -0.07 | | **-1.35** | **-2.78** |
|  |  | **6.84** | -0.01 | | -0.28 | **-0.93** | -0.14 | | **-0.77** | **-1.59** |
|  |  | **4.60** | -0.07 | | -0.36 | **-0.84** | -0.11 | | **-1.31** | **-3.02** |
|  |  | **6.33** | -0.07 | | -0.17 | **-0.61** | -0.13 | | **-0.66** | **-1.22** |
|  |  | **2.30** | -0.13 | | -0.12 | **-2.30** | -0.28 | | **-1.12** | **-2.30** |
|  | **median** | **5.05** | -0.07 | | -0.20 | **-0.89** | -0.13 | | **-0.95** | **-1.95** |

**Suppl. table 4** Percentage changes in median log HBV-RNA level after storage for 6, 48 or 169 h at 4, 25, and 42°C, respectively compared to baseline. Changes in concentration > log 0.5 are highlighted in black.

|  | |  | | **Whole blood** | | | | | **Supernatant** | | | | | |
| --- | --- | --- | --- | --- | --- | --- | --- | --- | --- | --- | --- | --- | --- | --- |
|  |  | | **BL**  **(log_10_ cp/mL)** | **6 h** | **48 h** | | **169 h** | | | **6 h** | **48 h** | | **169 h** | |
| **4°C** |  | |  |  |  | |  | | |  |  | |  | |
| **Plasma** | **> 10 – 100 copies/mL** | | **1.19** | -25.21% | -11.76% | | -11.76% | | | -6.72% | -13.44% | | 4.20% | |
|  | **> 100 copies/mL** | | **5.01** | 0.00% | 0.34% | | -0.20% | | | 0.20% | -0.80% | | -0.80% | |
| **Serum** | **> 10 – 100 copies/mL** | | **1.27** | -9.45% | -11.81% | | **-42.52**% | | | **-58.27**% | **-47.24**% | | -11.02% | |
|  | **> 100 copies/mL** | | **5.05** | -0.59% | 0.59% | | -0.40% | | | -0.20% | -0.80% | | -1.00% | |
| **25°C** |  | |  |  |  | |  | | |  |  | |  | |
| **Plasma** | **> 10 – 100 copies/mL** | | **1.19** | -10.92% | -9.24% | | **-47.90**% | | | 9.24% | -11.76% | | 11.76% | |
|  | **> 100 copies/mL** | | **5.01** | -0.40% | -2.20% | | -1.00% | | | -1.20% | -1.40% | | -2.80% | |
| **Serum** | **> 10 – 100 copies/mL** | | **1.27** | -4.72% | -21.25% | | **-85.83**% | | | -7.09% | -9.45% | | -20.47% | |
|  | **> 100 copies/mL** | | **5.05** | 0.00% | -1.58% | | -2.57% | | | -0.40% | -2.38% | | -6.14% | |
| **42°C** |  | |  |  |  | |  | | |  |  | |  | |
| **Plasma** | **> 10 – 100 copies/mL** | | **1.19** | -12.60% | **-95.80**% | | **-100.00**% | | | -9.24% | -11.00% | | -26.90% | |
|  | **> 100 copies/mL** | | **5.01** | -0.20% | -3.59% | | **-10.98**% | | | -1.34% | -5.79% | | **-18.16**% | |
| **Serum** | **> 10 – 100 copies/mL** | | **1.27** | **-47.24**% | **-100.0**% | | **-100.0**% | | | -33.07% | **-40.12**% | | **-64.57**% | |
|  | **> 100 copies/mL** | | **5.05** | -1.39% | -3.96% | | **-17.62**% | | | -2.57% | **-18.81**% | | **-38.61**% | |
|  |  | |  |  | |  | |  |  | | |  | |  |

**Supplement figures**


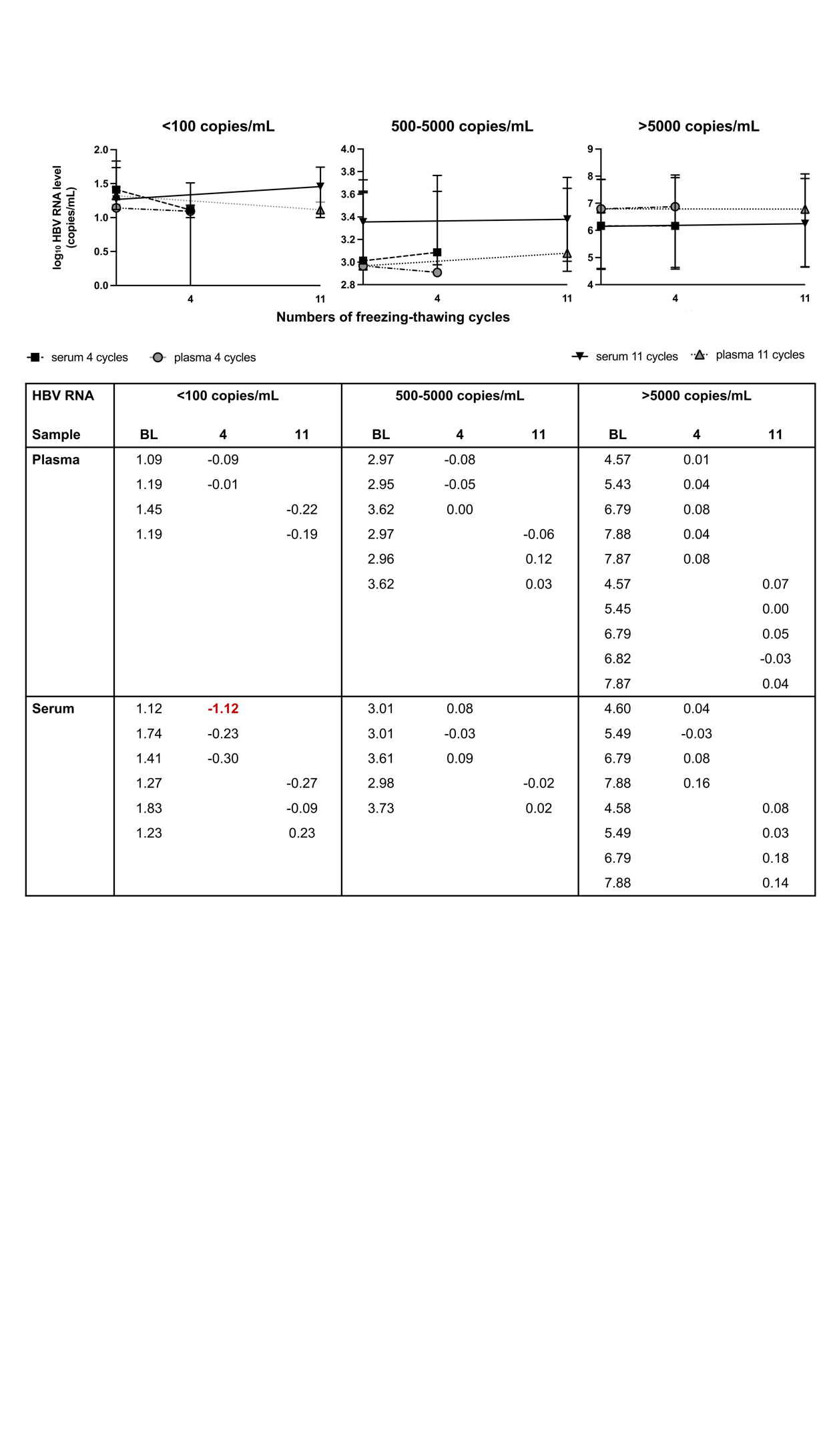


**Suppl. figure 1** Log_10_ HBV RNA (copies/mL) of serum and plasma samples and difference to baseline after 4 or 11 cycles of freezing and thawing. Numbers represent individual HBV RNA values (log_10_) at BL and after either 4 or 11 cycles of freezing and thawing.
